# A Cross‐Cultural Comparison of *ICD‐11* Complex Posttraumatic Stress Disorder Symptom Networks in Austria, the United Kingdom, and Lithuania

**DOI:** 10.1002/jts.22361

**Published:** 2019-01-28

**Authors:** Matthias Knefel, Brigitte Lueger‐Schuster, Jonathan Bisson, Thanos Karatzias, Evaldas Kazlauskas, Neil P. Roberts

**Affiliations:** ^1^ Faculty of Psychology University of Vienna Vienna Austria; ^2^ School of Medicine Cardiff University Cardiff UK; ^3^ School of Health & Social Care Edinburgh Napier University Edinburgh UK; ^4^ Rivers Centre for Traumatic Stress NHS Lothian Edinburgh Edinburgh UK; ^5^ Department of Clinical and Organizational Psychology Vilnius University Vilnius Lithuania; ^6^ Cardiff & Vale University Health Board Cardiff UK

## Abstract

The 11th revision of the World Health Organization's *International Classification of Diseases* (*ICD‐11*) includes a new disorder, complex posttraumatic stress disorder (CPTSD). The network approach to psychopathology enables investigation of the structure of disorders at the symptom level, which allows for analysis of direct symptom interactions. The network structure of *ICD‐11* CPTSD has not yet been studied, and it remains unclear whether similar networks replicate across different samples. We investigated the network models of four different trauma samples that included a total of 879 participants (*M* age = 47.17 years, *SD* = 11.92; 59.04% women) drawn from Austria, Lithuania, and Scotland and Wales in the United Kingdom. The International Trauma Questionnaire was used to assess symptoms of *ICD‐11* CPTSD in all samples. The prevalence of PTSD and CPTSD ranged from 23.7% to 37.3% and from 9.3% to 53.1%, respectively. Regularized partial correlation networks were estimated and the resulting networks compared. Despite several differences in the symptom presentation and cultural background, the networks across the four samples were considerably similar, with high correlations between symptom profiles (ρs = .48–.87), network structures (ρs = .69–.75), and centrality estimates (ρs = .59–.82). These results support the replicability of CPTSD network models across different samples and provide further evidence about the robust structure of CPTSD. The most central symptom in all four sample‐specific networks and the overall network was “feelings of worthlessness.” Implications of the network approach in research and practice are discussed.

The 11th revision of the World Health Organization's *International Classification of Diseases* (*ICD‐11*) has recently been published (First, Reed, Hyman, & Saxena, 2015). Within the classification of trauma‐ and stress‐related disorders, the *ICD‐11* introduced a new diagnostic category, *complex posttraumatic stress disorder* (CPTSD), as a sibling disorder to posttraumatic stress disorder (PTSD). In the *ICD‐11*, CPTSD describes a symptom profile that can arise following any traumatic event but is typically associated with exposure to multiple or repeated adverse events, including child abuse, torture, and severe domestic violence (Maercker et al., 2013). Individuals who present with CPTSD suffer from the *ICD‐11* PTSD symptoms of reexperiencing, avoidance, and sense of threat as well as three additional clusters of symptoms—affect dysregulation, negative self‐concept, and difficulties in relationships—which are described collectively as “disturbances in self‐organization” (DSO). This newly included disorder has been subjected to research, and an increasing number of studies support its clinical utility (see Brewin et al., 2017 for an extensive review). In the current study, we used network analysis to investigate the interaction between CPTSD symptoms and the robustness of the CPTSD network structure in four different samples from four European countries.

The network approach to psychopathology has gained attention in recent years (Borsboom, 2017). This approach defines mental disorders as sets of causally interacting symptoms. This definition differs from the *latent variable model*, which is the typical model of mental disorders. In the latent variable model, disorders are defined as a latent entity that is not directly observable and can only be assessed indirectly by the measurement of symptoms. The symptoms are thus reflective of the disorder (Borsboom, 2008). By comparison, the network approach assumes that symptoms can be initially triggered by external factors, such as a traumatic event in the case of PTSD. Once triggered, a symptom will likely and directly lead to other symptoms (e.g., flashbacks may lead to sense of threat which in turn may lead to concentration problems) and maybe even activate negative symptom loops (Cramer et al., 2016). In this model, the observable symptoms alone are sufficient to constitute the disorder (Borsboom & Cramer, 2013). Network analysis is a method that allows visualization of the structure of symptom associations and identifies symptoms that are particularly central in the network. These symptoms are arguably the most important symptoms in a disorder.

To our knowledge, no study thus far has used a network analytical approach to investigate the *ICD‐11* formulation of CPTSD. Knefel, Tran, and Lueger‐Schuster (2016) used a network approach to investigate the comorbidity of *ICD‐11* CPTSD and borderline personality disorder in a sample of adult survivors of child maltreatment and found that “feelings of worthlessness” was the most central CPTSD symptom in the resulting network. Two studies that evaluated the network properties of PTSD as defined in the fifth edition of the *Diagnostic and Statistical Manual of Mental Disorders* (*DSM‐*5) found that “negative trauma related emotions” and “reactivity to cues” were among the most central symptoms in both networks (Armour, Fried, Deserno, Tsai, & Pietrzak, 2017; Spiller et al., 2017). The network approach is still relatively new in the study of psychopathology, and it is not yet clear how robust the results from single data sets are and whether they will replicate and generalize to other samples (Epskamp, Borsboom, & Fried, 2017; Fried et al., 2018). For example, although “detachment” was among the most central symptoms in one of the aforementioned studies (Armour et al., 2017), this was not the case in the other study, wherein “self‐destructive or reckless behavior” was instead central (Spiller et al., 2017). Fried et al. (2018) addressed this issue and compared the network structures of PTSD as defined in the fourth edition of the *DSM* (DSM‐IV) across four samples. The authors found good support for the replicability of network models. Therefore, we followed this approach and analyzed the network models of *ICD‐11* CPTSD in four different samples from four different countries: Austria, Lithuania, and the United Kingdom (Scotland and Wales). Our aims were to (a) investigate the network structure in four different samples using an estimation procedure that took similarities between the samples into account, (b) find central symptoms within the networks, (c) test the accuracy of these estimations, and (d) compare the networks across the four samples.

## Method

### Participants and Procedure

Participants from four traumatized samples were included in our analysis (*N* = 879). The mean age for the total sample was 47.17 years (*SD* = 11.92, range: 18–87 years), and the majority of participants in the sample were women (59.04%). Table 1 depicts the characteristics of each sample.

**Table 1:** Descriptive Sample Characteristics

				Age				
Sample	Description	Country	Sample Size (*N*)	*M*	*SD*	% Women	% *ICD‐11* PTSD^a^	% *ICD‐11* CPTSD^a^
1	Survivors of child maltreatment	Austria	220	57.90	9.55	40.0	37.3	17.3
2	Primary mental health care patients	Lithuania	280	39.48	13.35	77.5	27.9	9.3
3	Trauma center patients	Scotland (UK)	193	40.56	12.30	65.1	37.0	53.1
4	Primary and secondary mental health service users	Wales (UK)	186	48.40	12.32	47.3	23.7	41.9

*Note. ICD‐11 = International Classification of Diseases* (11th rev.); PTSD = posttraumatic stress disorder; CPTSD = complex posttraumatic stress disorder.

a PTSD and CPTSD rates are based on self‐report.

The first sample consisted of 220 Austrian adults who were survivors of child maltreatment during foster care placement. Data were collected as part of the Vienna Institutional Abuse Study (Lueger‐Schuster et al., 2017). Child maltreatment was assessed with the Childhood Trauma Questionnaire (CTQ; Bernstein et al., 2003) and traumatic life events in adulthood were assessed with the Life Events Checklist for *DSM‐5* (LEC‐5; Weathers et al., 2013). All participants in this sample lived in institutional foster care during their childhood and experienced maltreatment during this time. Endorsement rates for any item (i.e., a score of greater than 1) from the CTQ subscales indicated that any experience of childhood trauma was very high: 100.00% for emotional neglect, 99.5%, for physical neglect, 99.5% for emotional abuse, 98.2% for physical abuse, and 70.0% for sexual abuse. The mean number of adult traumatic life event types participants in this sample experienced was 5.65 (*SD* = 3.09). More than one‐third (37.3%) of the sample fulfilled the proposed criteria for *ICD‐11* PTSD and another 17.3% fulfilled the proposed criteria for *ICD‐11* CPTSD. The study was approved by the Institutional Review Board of the University of Vienna and all participants gave full written informed consent.

The second sample consisted of 280 adult primary mental health care patients in Lithuania (Kazlauskas, Gegieckaite, Hyland, Zelviene, & Cloitre, 2018). Participants were recruited at primary mental health centers, outpatient mental health clinics and hospitals, private clinical psychologists’ practice, and addiction rehabilitation centers. Lifetime traumatic events were assessed using the LEC‐5, and individuals in this sample reported on average 4.60 (*SD* = 2.55) types of lifetime traumatic experiences. The prevalence of proposed *ICD‐11* PTSD and CPTSD were 27.9% and 9.3%, respectively. This study was approved by the Vilnius University Institutional Psychological Research Ethics Committee.

The third sample consisted of 193 individuals who were referred for psychological therapy to a National Health Service (NHS) trauma center in Scotland (Karatzias et al., 2016). Cases of traumatization that occurred in childhood, adulthood, or both were referred to the service. Child maltreatment was assessed using the CTQ, and adult life events were assessed using the LEC‐5. Endorsement rates for any item (i.e., a score greater than 1) from the CTQ subscales indicated that childhood trauma was frequent: 84.6% for emotional abuse, 63.8% for physical abuse, 53.3% for sexual abuse, 79.8 % for emotional neglect, and 68.6% for physical neglect. The mean number of lifetime traumatic event types participants in this sample experienced was 5.00 (*SD* = 2.48). The prevalence of proposed *ICD‐11* PTSD and CPTSD were 37% and 53.1%, respectively. The study was approved by the United Kingdom's National Research Ethics Service.

The fourth sample consisted of 186 adults from Wales (United Kingdom) who were recruited to the National Centre for Mental Health cohort via primary and secondary mental health services, specialist veterans’ services, a specialist civilian trauma service, and via social media (Hyland et al., 2017). Adult life events were assessed using an adapted version of the LEC‐5, which included additional items for childhood sexual and physical abuse. Nearly half of participants in the sample (47.9%) reported physical or sexual child abuse, and the average number of lifetime traumatic experience types was 6.90 (*SD* = 3.83). In this sample, 23.4% fulfilled criteria for proposed *ICD‐11* PTSD and 41.5% for CPTSD. The study received ethical approval from the United Kingdom's National Research Ethics Service.

### Measures

#### *ICD‐11* PTSD and CPTSD

All four studies used the International Trauma Questionnaire (ITQ; Cloitre, Roberts, Bisson, & Brewin, 2013) to assess the proposed symptoms of *ICD‐11* PTSD and CPTSD. The ITQ is a self‐report measure of the *ICD‐11* symptoms of PTSD and CPTSD (see Karatzias et al., 2018 for a recent review on the psychometric properties of the ITQ). There are six items that measure three PTSD clusters: reexperiencing in the here and now; deliberate avoidance of traumatic reminders (internal or external), and a sense of current threat. Sixteen items measure the three DSO factors: Affective dysregulation (nine items covering both hyperactivation [five items] and hypoactivation [four items]); negative self‐concept (four items); and difficulties in relationships (three items). Respondents are instructed to respond in relation to how much they have been bothered by each symptom in the past month, and are instructed to answer the DSO items in relation to how they typically feel, think about themselves, and relate to others. All items are answered on a 5‐point Likert scale that ranges from 0 (*not at all*) to 4 (*extremely*). Diagnostic criteria for PTSD require a score of 2 (*moderately*) or more for at least one of two symptoms from each of the three PTSD clusters. A CPTSD diagnosis requires that an individual meets the PTSD criteria and endorses each DSO symptom cluster at a moderate level of severity, defined as summed score that equals a score of 2 or greater for each of the items in the cluster: a summed total score of 10 or higher for the five items that reflect hyperactivation or a summed total score of 8 or higher for the four items that reflect hypoactivation; a summed total score 8 or higher for the four items that reflect negative self‐concept; and a summed total score 6 or higher for the three items that reflect difficulties in relationships. The *ICD‐11* requires the presence of functional impairment associated with both sets of symptoms for a diagnosis of PTSD and CPTSD. However, functional impairment was not assessed in the current study; therefore, diagnostic rates are based on symptom criteria alone. The *ICD‐11* taxonomic structure means that an individual can only be diagnosed with PTSD or CPTSD, not both. The studies performed in Scotland and Wales used the English version of the ITQ (Cloitre et al., 2013), the Austrian study used the German version (Knefel, Lueger‐Schuster, & Maercker, 2013), and the Lithuanian study used the Lithuanian version of the ITQ (Kazlauskas et al., 2018). All versions have demonstrated good psychometric properties in previous research (English Version: Karatzias et al., 2016; German version: Knefel et al., 2016; Lithuanian version: Kazlauskas et al., 2018). The Cronbach's alpha values for the total scale were good in all samples (Cronbach's αs = .91–.94); Cronbach's alpha for the total sample was .95.

### Data Analysis

We followed the statistical procedure described by Fried et al. (2018) and conducted four steps of analysis: network estimation, network inference, network stability, and network comparison. We used the R statistical environment (R Core Team, 2016) for all analyses and the package qgraph (Epskamp, Cramer, Waldorp, Schmittmann, & Borsboom, 2012) to visualize all networks. The R code for our analyses can be found in the Supplementary Materials and the correlations matrices are available upon request.

#### Missing values

There were only a few ITQ missing values in the four data sets (range: 0–15 missing values). We retained all participants for the network analysis and used pairwise complete observations to estimate the correlations among the symptoms.

#### Network estimation

Symptom networks consist of nodes, which represent symptoms, and edges, which represent the pairwise associations between two nodes. Within the results, symptom nodes are referred to as *short codes*; please see Table 2 for corresponding full symptom names. We estimated Gaussian graphical models (GGM) for pairwise association parameters between all nodes. In the GGM, edges can be understood as conditional dependence associations among symptoms: If two symptoms are connected in the resulting graph, they are dependent after controlling for all other symptoms. Symptoms that are not connected via an edge are conditionally independent. With 22 symptom nodes, 231 pairwise association parameters were estimated. The estimation of so many parameters is likely to lead to a number of spurious connections; we thus controlled for these false positives by using the least absolute shrinkage and selection operator (LASSO; Friedman, Hastie, & Tibshirani, 2008), which sets very small edges to zero. This procedure employs a regularization technique that conservatively identifies only the relevant edges and accurately discovers the underlying network structure (van Borkulo et al., 2014). More details on these estimation techniques, including a tutorial, are available elsewhere (Epskamp & Fried, 2017). Because PTSD symptoms can be considered ordered‐categorical, we based the estimation of the 22‐item networks on the polychoric correlation among symptoms.

**Table 2:** Means and Standard Deviations of Symptoms

		Austria		Lithuania		Scotland		Wales	
Symptom	Short code	*M*	*SD*	*M*	*SD*	*M*	*SD*	*M*	*SD*
Distressing dreams	RE1	1.28	1.56	1.03	1.21	2.66	1.30	2.24	1.44
Intrusive recollections	RE2	1.67	1.55	1.27	1.36	2.48	1.39	2.30	1.41
Internal avoidance	AV1	1.84	1.54	1.54	1.40	2.92	1.06	2.67	1.25
External avoidance	AV2	1.65	1.56	1.52	1.45	3.03	1.08	2.72	1.35
Hypervigilance	TH1	2.45	1.61	1.23	1.28	3.07	1.21	2.69	1.32
Exaggerated startle response	TH2	1.74	1.58	1.62	1.36	2.89	1.25	2.53	1.38
Heightened emotional reactivity	AD1	2.41	1.37	1.86	1.10	2.66	1.16	2.52	1.14
Long‐time upset	AD2	2.29	1.50	1.95	1.08	2.71	1.06	2.78	1.14
Emotional vulnerability	AD3	2.83	1.33	2.28	1.18	2.69	1.18	2.69	1.23
Anger	AD4	1.48	1.49	1.53	1.25	1.79	1.45	1.69	1.48
Reckless behavior	AD5	0.85	1.26	0.88	1.15	1.20	1.46	1.28	1.38
Emotional numbing	AD6	1.39	1.51	0.92	1.13	2.61	1.25	2.42	1.33
Inability experiencing positive emotions	AD7	1.49	1.55	1.10	1.22	2.24	1.35	2.18	1.43
Derealization	AD8	1.87	1.62	1.19	1.22	2.83	1.25	2.41	1.35
Depersonalization	AD9	1.59	1.62	1.07	1.22	2.22	1.52	2.01	1.51
Feelings of failure	NSC1	0.83	1.21	1.10	1.27	2.68	1.41	2.28	1.42
Feelings of worthlessness	NSC2	0.89	1.35	1.04	1.3	2.49	1.48	2.14	1.51
Feelings of shame	NSC3	1.10	1.36	1.09	1.24	2.65	1.37	2.35	1.42
Feelings of guilt	NSC4	1.66	1.44	1.91	1.22	2.85	1.26	2.70	1.26
Feeling distant or cut off from others	DR1	1.23	1.39	1.42	1.25	2.78	1.16	2.55	1.32
Difficulties feeling close to others	DR2	1.68	1.61	1.23	1.22	2.49	1.35	2.23	1.40
Avoidance of relationships	DR3	1.65	1.66	1.13	1.24	2.26	1.55	1.94	1.56
Total Mean^a^		1.63	0.89	1.36	0.78	2.54	0.77	2.29	0.90

*Note*. Symptoms assessed using the International Trauma Questionnaire.

a *t* tests comparing total means: Lithuania < Austria < Wales < Scotland, *t*s(329.60–432.96) = 2.74–16.16, *p* < .001 to *p* = .007.

The aim of our study was to compare the networks of four different samples. Assuming the networks of the four samples are identical, the best estimation would be a single GGM on the combined sample. However, as described by Fried et al. (2018), this would neglect that the true networks might differ between the samples. The complementary approach would be to estimate each network separately for all four samples. This would allow for a comparison of the networks across samples, but it would also result in poorer estimates if the networks were, in fact, identical. Especially given the relatively small sample sizes in our study, this would be associated with a relevant loss of power. The joint estimation of different graphical models using a recently developed network estimation technique, the fused graphical lasso (FGL), addresses these issues (Costantini et al., 2017). The FGL is a valid method that can lead to a more accurate estimation of network structures than estimating networks individually (Costantini et al., 2017; Danaher, Wang, & Witten, 2014).This method comes close to estimating networks independently, if the true networks are distinct and exploiting similarities would not improve model fit. Thus, true differences are allowed to emerge. This property makes the FGL a good method for estimating networks in different groups (Richetin, Preti, Costantini, & Panfilis, 2017) and we therefore used the FGL in our study. We used the R package EstimateGroupNetwork (Costantini & Epskamp, 2017) for network estimation employing the *k*‐fold cross‐validation for parameter selection as implemented in the package and selected the default value for *k* = 10.

#### Network inference

We used two parameters to describe the connectedness of each node in the four jointly estimated networks: the centrality index *node strength* and the *predictability* of each node. Strength refers to the sum of all edges connected to a specific node (Opsahl, Agneessens, & Skvoretz, 2010). Other centrality parameters, *betweenness* and *closeness*, are reported in the Supplementary Materials because they could not be estimated reliably in the present manuscript, which has also been suggested in recent research (Epskamp et al., 2017). Predictability refers to the estimated shared variance of each node with all of its neighbors (Haslbeck & Fried, 2017). We estimated predictability using the R “mgm” package (Haslbeck, 2015). Strength and predictability both provide information on the connectedness of each node within the symptom network. Whereas strength can be regarded as a relative metric, predictability is an absolute measure of connectedness. Predictability can be understood as an upper boundary for each node to possible influence by its neighboring nodes. Assuming that all connections go toward this node, predictability quantifies how much influence could be exerted on this node through intervening on all its neighbors.

#### Network stability

Network stability estimation has only recently been introduced (Epskamp et al., 2017). At the moment, there is no method available to test the stability of jointly estimated networks. We thus followed the procedure by Fried et al. (2018) and examined the stability of the individual networks. We used the R “bootnet” package (Epskamp, 2015) and bootstrapped 95% confidence intervals around the edge weights, estimated the correlation‐stability coefficient for centrality metrics (ranging from 0 to 1; values above .25 imply moderate stability and above .50 imply strong stability; Epskamp et al., 2017), and computed the edge‐weights difference test and the centrality difference test for each network.

#### Network comparison

To obtain an index of the degree of similarity across the samples, we correlated the edge weights across the four networks (Rhemtulla et al., 2016). We then used the R “NetworkComparisonTest” package (NCT; van Borkulo et al., 2017) for several comparisons. First, we used an overall test to investigate whether all edges in all pairs of networks were identical. Second, we applied post‐hoc comparisons using the Holm–Bonferroni correction for multiple testing to estimate the number of edges that differed between each pair of networks. Third, we tested whether the sum of all edge weights within each network (i.e., global strength) differed across the networks. In a next step, we averaged the edge weights across the four networks and visualized the resulting cross‐sample network. Finally, we constructed a network to visualize the differences and similarities of the edges across the samples using the standard deviation of each edge across the four networks (Rhemtulla et al., 2016).

## Results

### Descriptive Statistics

The average level of symptom distress differed between the four samples (Table 2). Scottish trauma center patients reported the highest level of distress, followed by Welsh primary and secondary mental health–service users, Austrian survivors of child maltreatment during foster care, and Lithuanian primary mental health care patients. The symptom profiles were relatively similar across the four samples: Spearman correlations between the symptom profiles ranged from ρ = .48 (Austrian and Scottish samples) to ρ *=* .87 (Scottish and Welsh samples). The mean symptom profile correlation was ρ *=* .64. The mean values of the single symptoms ranged from 0.83, for “feelings of failure” in the Austrian sample, to 3.07, for “hypervigilance” in the Scottish sample. “Emotional vulnerability” was among the most prevalent symptoms in all samples except for the Scottish sample whereas “reckless behavior” was among the least prevalent symptoms across all samples.

### Network Estimation

**Figure 1:**
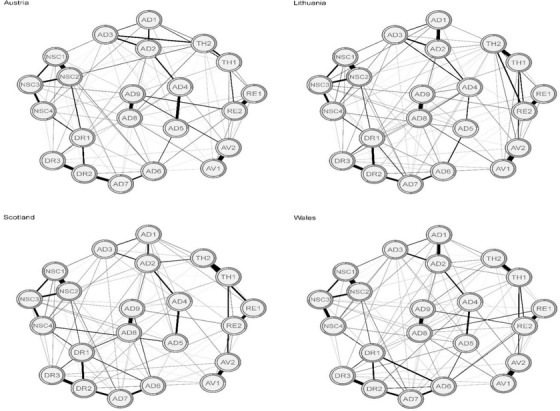
Regularized partial correlation networks across four data sets of traumatized individuals. Edge thickness represents the degree of association, solid edges indicate positive relations, and dashed edges indicate negative relationships. The gray area in the rings around the nodes depicts predictability (the variance of a given node explained by all its neighbors). RE1 = distressing dreams; RE2 = intrusive recollections; AV1 = internal avoidance; AV2 = external avoidance; TH1 = hypervigilance; TH2 = exaggerated startle response; AD1 = heightened emotional reactivity; AD2 = long‐time upset; AD3 = emotional vulnerability; AD4 = anger; AD5 = reckless behavior; AD6 = emotional numbing; AD7 = inability experiencing positive emotions; AD8 = derealization; AD9 = depersonalization; NSC1 = feelings of failure; NSC2 = feelings of worthlessness; NSC3 = feelings of shame; NSC4 = feelings of guilt; DR1 = feeling distant or cut off from others; DR2 = difficulties feeling close to others; DR3 = avoidance of relationships.

Figure 1 depicts the results of the four jointly estimated networks. In the Austrian, Lithuanian, Scottish, and Welsh samples, 108, 113, 107, and 117 of all possible 231 edges (46.8%, 48.9%, 46.3%, and 50.6%, respectively) were estimated to be above zero, which means that the symptoms had substantial connections to each other. The visual inspection of the four networks shows many consistent edges, such as strong connections between distressing dreams (RE1) and intrusive recollections (RE2), difficulties feeling close to others (DR2) and avoidance of relationships (DR3), internal avoidance (AV1) and external avoidance (AV2), feelings of failure (NSC1) and feelings of worthlessness (NSC2), and derealization (AD8) and depersonalization (AD9; see Table 2 for all symptom names). Other edges differed between the networks, such as (a) the edge between hypervigilance (TH1) and exaggerated startle response (TH2), which was strong in three networks but rather weak in the Austrian network; (b) the edge between anger (AD4) and reckless behavior (AD5), which was rather strong in all networks except for the Lithuanian network; and (c) the edge between RE1 and TH2, which was relatively strong in the Scottish network but rather weak in all other networks. The edge between emotional vulnerability (AD3) and AD5 was negative in the Austrian and the Welsh networks but it was fixed to zero by the LASSO in the other two networks.

### Network Inference

**Figure 2:**
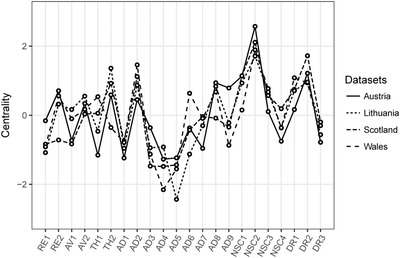
Standardized node strength centrality of the 22 complex posttraumatic stress disorder symptoms across four clinical data sets of traumatized patients receiving treatment. RE1 = distressing dreams; RE2 = intrusive recollections; AV1 = internal avoidance; AV2 = external avoidance; TH1 = hypervigilance; TH2 = exaggerated startle response; AD1 = heightened emotional reactivity; AD2 = long‐time upset; AD3 = emotional vulnerability; AD4 = anger; AD5 = reckless behavior; AD6 = emotional numbing; AD7 = inability experiencing positive emotions; AD8 = derealization; AD9 = depersonalization; NSC1 = feelings of failure; NSC2 = feelings of worthlessness; NSC3 = feelings of shame; NSC4 = feelings of guilt; DR1 = feeling distant or cut off from others; DR2 = difficulties feeling close to others; DR3 = avoidance of relationships.

The standardized strength centrality estimates are presented in Figure 2. These estimates were very similar across the four networks, with Spearman correlations ranging from ρ = .59 in the Austrian and Welsh samples to ρ = .82 in the Scottish and Lithuanian samples. We found that NSC2 had the highest‐strength metric across all samples, and DR2 had relatively high values in all networks. The strength of AD4 and AD5 were among the lowest in all samples. The strength metrics of TH1, TH2, emotional numbing (AD6), and AD9 had the highest cross‐sample variation. To evaluate a possible bias (Terluin, de Boer, & de Vet, 2016), we correlated the strength centrality estimates with the variance of each symptom and found a small average correlation of*r* = .14.

The average predictability of the nodes is graphically presented in Figure 1, and it ranged from 0.47 (Austrian and Scottish samples) to 0.60 (Welsh sample), with a total mean of 0.52. This means that, on average, 47% to 60% of the variation of each symptom could be explained by its neighboring symptoms. Strength and predictability were closely related (correlations from ρs = .80–.90), which reflects their conceptual similarity.

### Network Stability

There are no clear boundaries with which to interpret the results of the stability analyses. The confidence intervals around the edge weights were moderately large, which indicates a moderate accuracy of the network estimation. The correlation stability coefficient for the strength centrality metric was above the suggested .50 threshold for strong stability (Epskamp et al., 2017) for the Scottish sample (.52) and above the suggested threshold of .25 for moderate stability for the other three samples (Austrian sample, .44; Lithuanian sample, .44; Welsh sample, .36). The results of the stability analyses are detailed in the Supplementary Materials.

### Network Comparison

Spearman correlations of the edge weights among the samples ranged from ρ = .69 (Austrian and Scottish samples) to ρ = .75 (Austrian and Lithuanian samples), which indicated strong similarities. The NCT is an overall test of network similarity. We compared all six pairs of networks and found that the network identified for the Austrian sample differed from those identified in all other samples, whereas the networks identified among the Welsh and the Lithuanian participants differed significantly from each other, *p*s = .004–.049. We then used a post‐hoc test to compare all edges among the networks and found only one significantly differing edge, between TH1 and TH2, in the comparison of the Austrian and the Scottish networks; no other edge was found to significantly differ among all networks. The global strength of the networks, which is a measure of the overall connectivity within a network, was 9.66 for the Austrian network, 9.89 for the Lithuanian network, 9.41 for the Scottish network, and 10.13 for the Welsh network. The NCT showed significant differences only for the comparison of the overall connectivity between the Scottish and Welsh networks as well as the Scottish and Lithuanian networks. Collectively, these results therefore suggest a strong similarity among the networks.

**Figure 3:**
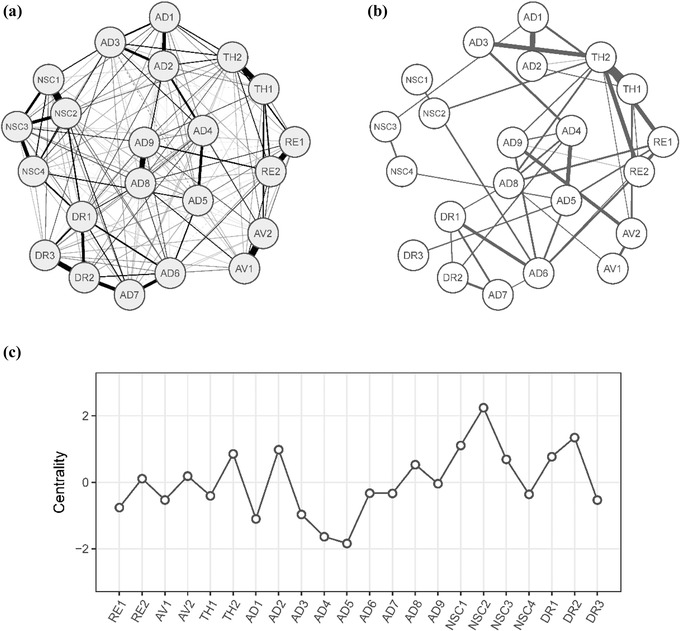
Network analysis in the combined data set. Cross‐sample network (*n* = 879; Panel A) depicts the average of the four individual networks; solid edges indicate positive associations, and dashed edges indicate negative relationships. In the cross‐sample variability network (Panel B), each edge depicts the standard deviation of this edge across the four networks. Panel C shows standardized node strength centrality for the cross‐sample network. RE1 = distressing dreams; RE2 = intrusive recollections; AV1 = internal avoidance; AV2 = external avoidance; TH1 = hypervigilance; TH2 = exaggerated startle response; AD1 = heightened emotional reactivity; AD2 = long‐time upset; AD3 = emotional vulnerability; AD4 = anger; AD5 = reckless behavior; AD6 = emotional numbing; AD7 = inability experiencing positive emotions; AD8 = derealization; AD9 = depersonalization; NSC1 = feelings of failure; NSC2 = feelings of worthlessness; NSC3 = feelings of shame; NSC4 = feelings of guilt; DR1 = feeling distant or cut off from others; DR2 = difficulties feeling close to others; DR3 = avoidance of relationships.

Thus, as a final step, we estimated a network for the total sample of 879 traumatized patients. The network graph of this cross‐sample network is displayed in Figure 3A. As might be expected, the structure of this network was similar to the structures of the four jointly estimated networks: It showed strong connections between RE1 and RE2, NSC1 and NSC2, AV1 and AV2, TH1 and TH2, and AD8 and AD9. We found that NSC2 had the highest strength, followed by DR2, NSC1, long‐time upset (AD2), TH2, and feeling distant or cut off from others (DR1). The least central symptom in this network was AD5 (Figure 3C). Figure 3B shows a network that visualizes the differences and similarities of the edges across the samples. In this network, the differences of each edge between any two symptoms across the four networks is illustrated as an edge: Strong edges mean strong variation of the respective edge across the four samples. The largest variation could be observed between TH1 and TH2 (*SD* = 0.13), heightened emotional reactivity (AD1) and AD2 (*SD* = 0.10), and TH1 and AD3 (*SD* = 0.09). For most edges, the internetwork variation was negligibly small.

## Discussion

To our knowledge, this was the first investigation of the *ICD‐11* CPTSD network structure. We jointly estimated four networks in four trauma samples that varied in their European cultural background, demographic characteristics, trauma experiences, and symptom severity. Symptoms of *ICD‐11* CPTSD were assessed with the same instrument, the ITQ, in all samples, thus ruling out possible assessment differences as bias for the comparison. In summary, we found that even though the severity of symptom distress differed across samples, the symptom profiles correlated strongly across the samples. In the jointly estimated networks, about half of all possible edges was estimated to be non‐zero. The visual impression that the four networks were highly similar was cautiously supported by the formal network comparison, which revealed only minor differences. The most central symptom in all four sample specific networks and the overall network was NSC2 (feelings of worthlessness). The results were at least moderately robust and accurate as shown by the stability analyses. In all four sample‐specific networks, as well as in the overall network, the connections between some symptoms were very strong, including both reexperiencing symptoms, RE1 (distressing dreams and RE2 (intrusive recollections); both avoidance symptoms, AV1 (internal avoidance) and AV2 (external avoidance); both dissociative symptoms, AD8 (derealization) and AD9 (depersonalization); two symptoms of the DSO negative self‐concept domain, NSC1 (feelings of failure) and NSC2 (feelings of worthlessness); and two symptoms of the DSO difficulties in relationships domain, DR2 (difficulties feeling close to others) and DR3 (avoidance of relationships). The largest variation in symptom connectivity between the samples was for the connections of the two sense‐of‐threat symptoms, TH1 (hypervigilance) and TH2 (exaggerated startle response); and two symptoms of the DSO affect dysregulation domain, AD1 (heightened emotional reactivity) and AD2 (long‐time upset).

This study supports the robustness and replicability of network models because we found a relatively stable pattern of associations across four different samples, which is in contrast to recent publications that have questioned whether these models would generalize and replicate in different samples (see Borsboom et al., 2017 for an overview). The present study provided evidence that this type of model can be replicated and thus supports the findings of Fried et al. (2018). Although Fried and colleagues (2018) used *DSM‐IV* PTSD symptoms in their analysis, we followed their analytical strategy and can thus compare our results on a methodological level. In both studies, the cross‐sample networks had high similarity, as shown by the intercorrelation of the edge weights and the strength centrality estimates. The formal network comparison test did not detect large differences between samples; however, the sample size in the current study limited the sensitivity of this test. The four samples in our study differed widely with respect to prevalence rates of *ICD‐11* PTSD and CPTSD, which ranged from 23.7% to 37.3% for PTSD and 9.3% to 53.1% for CPTSD, and we found similar networks across these samples with different levels of symptom burden. This result gives preliminary evidence for the replicability of CPTSD network models across different populations.

The connections of several symptoms in the networks were considerably stronger than those of other symptoms. We found that any two symptoms that were among those with the strongest connections were from the same symptom domain: reexperiencing, difficulties in relationships, avoidance, negative self‐concept, and affect dysregulation (dissociation). This supports the conceptual similarity of these symptoms within their respective domains and the proposed factor structure of *ICD‐11* CPTSD (Kazlauskas et al., 2018; Shevlin et al., 2017). This result is similar to the findings that have been reported in factor analytical studies, in which symptoms within a factor are strongly related to each other (e.g. Hyland et al., 2017). This is not surprising because under certain conditions, network models and factor models are mathematically equivalent (Kruis & Maris, 2016) and both are based on the correlation matrix of the data. However, conceptual assumptions that underlie these models differ and the network approach emphasizes the mutual interaction between symptoms. Given its similarities to the factor model, the network approach does not introduce a completely new way of modeling associations of symptoms, but it provides novel possibilities to investigate the role of certain symptoms and points toward the dynamic and complex symptom interplay within mental disorders (Bringmann & Eronen, 2018). Although the theoretical explanation of statistical covariation of symptoms within a cluster in the factor model is the presence of a latent variable that causes the symptoms, the network approach suggests a direct interaction on symptom level. Notably, the network approach does not preclude the existence of a directly not observable variable, such as brain circuits, accounting for observable covariation on symptom level.

The most central symptom in all networks was NSC2 (feelings of worthlessness). This seems similar to prior results on the *DSM‐*5 network structure of PTSD, in which “negative trauma‐related emotions” showed the highest centrality (Armour et al., 2017). The second most central symptom, DR2 (difficulties feeling close to others [detachment]), was also found to be among the most central symptoms in the analyses by Fried et al. (2018). High centrality means that these symptoms have strong associations with neighboring symptoms. As our analysis was cross‐sectional, however, we could draw no conclusions regarding the directionality of these associations. It is possible that considering oneself as worthless is the consequence of many other symptoms, which seems plausible because symptom distress is usually associated with functional impairment (Maercker et al., 2013), which in turn could lead to a negative self‐concept that finds its expression in feelings of worthlessness. However, the opposite seems plausible as well: Feelings of worthlessness could lead to other negative self‐concept representations, which in turn could lead to difficulties in relationships and so on. We conclude that it seems most likely that a central symptom is bidirectionally related to its neighbors. The important question to this end is whether interventions that address central symptoms are more likely to lead to overall symptom relief than interventions that address other symptoms (Fried et al., 2018; Hofmann, Joshua, & McNally, 2016). The answer to this question depends on the actual causal direction, which could not be determined in our study. Nevertheless, it seems advisable to focus on central rather than on decentral nodes when planning interventions.

Finally, we think it is important to address the association between cross‐sectional between‐person networks and longitudinal within‐person networks. It is possible that a cross‐sectional network, such as the networks presented in this manuscript, significantly differs from an individual network consisting of one person's symptoms as measured over several time points. All conclusions drawn from our analyses should be interpreted in the light of a between‐person approach. Applying these results to predict the course of an individual within‐person network cannot be justified and future research should investigate these issues.

Despite the robust methodological design, this study had some limitations that need to be considered when interpreting the results. First, all studies used the ITQ to assess symptoms of CPTSD. Although it is a strength that symptoms were measured with the same instrument in all samples, the ITQ is a self‐report questionnaire and a clinician‐administered interview might provide more valid data on symptom burden. Second, there are likely to be similarities in the cultural backgrounds of the four samples even though all samples came from different regions of Europe. It is not clear whether our results would generalize to other traumatized populations, such as refugees, veterans, or populations from other areas of the world. Third, the size of the individual samples limited some of the analyses, especially the overall network comparison test. It is possible that larger sample sizes with more power would have detected differences that we missed. Fourth, the *ICD‐11* requires the presence of functional impairment associated with symptoms for a diagnosis of CPTSD. However, functional impairment was not assessed in the current study, and it is possible that considering only participants who report functional impairment would result in different networks. Finally, all data used in this study were cross‐sectional, limiting possible causal interpretations.

In conclusion, this study was the first that used network analysis to investigate the structure of *ICD‐11* CPTSD with state‐of‐the‐art methods. The similarity of the networks across the four samples supports the structure of CPTSD, which seems to represent a similar disorder across different cultural groups. Future research should investigate causality and the association between between‐person and within‐person networks as well as the hypothesis that targeting central symptoms leads to faster recovery than targeting decentral symptoms.

## Supporting information

Supporting Information

Figure S1. Stability analysis: Accuracy of edge weights. Bootstrapped confidence intervals (CIs) of the edge weights for the four individually estimated networks, derived from non‐parametric bootstrap (nBoot=1000) analyses using R‐package bootnet (Epskamp et al., 2017).
Figure S2. Stability Analysis: Centrality bootstrap. Correlation of the original centrality order with the order of centrality in subsets of the data.
Figure S3. Edge weights difference test. Black boxes represent significant differences between edge weights. The test does presently not correct for multiple testing.
Figure S4. Centrality difference test. Standardized centrality values are shown in the diagonal, black boxes represent significant differences centrality estimates. The test does presently not correct for multiple testing.
Figure S5. Centrality for the four independently estimated networks. Note that only strength had acceptable stability and thus the size of the other estimates should not be interpreted.
