# Supplementary material for: A Cross‐Cultural Comparison of ICD‐11 Complex Posttraumatic Stress Disorder Symptom Networks in Austria, the United Kingdom, and Lithuania
Source: J Trauma Stress. 2019 Jan 28;33(1):41–51. doi: 10.1002/jts.22361 (PMC7155025; doi:10.1002/jts.22361)
Supplement: Supplementary file 1 — Supporting Information [file JTS-33-41-s001.pdf]

```
###############################################################################################
#                                                                                             #
#                 Cross-cutural comparison of the network structure                           #
#                         of ICD-11 Complex PTSD symptoms                                     #
#                                                                                             #
#                              Knefel at al., 2018                                            #
#                    submitted to Journal of Traumatic Stress                                 #
#                                                                                             #
###############################################################################################


# ---------- Table of Contents -------------------------------------------------------
# ---------- 1. Load Libraries -------------------------------------------------------
# ---------- 2. Load & manipulate data -----------------------------------------------
# ---------- 3. Descriptives ---------------------------------------------------------
# ---------- 4. Estimate networks individually ---------------------------------------
# ---------- 5. Stability and accuracy for individually estimated networks ------------
# ---------- 6. Joint network estimation ---------------------------------------------
# ---------- 7. Centrality for 4 jointly estimated networks --------------------------
# ---------- 8. Network comparison tests for 4 jointly estimated networks -------------
# ---------- 9. Pool data into one full network --------------------------------------



# ------------------------------------------------------------------------------------
# ---------- 1. Load libraries -------------------------------------------------------
# ------------------------------------------------------------------------------------


#setwd("...")
#load libraries: qgraph, dplyr, EstimateGroupNetwork, bootnet, mgm, NetworkComparisonTest, lavaan


# ------------------------------------------------------------------------------------
# ---------- 2. Load & manipulate data -----------------------------------------------
# ------------------------------------------------------------------------------------


data1 <- read.table(...)
data2 <- read.table(...)
data3 <- read.table(...)
data4 <- read.table(...)

names <- c("RE1", "RE2", "AV1", "AV2", "TH1", "TH2", "AD1", "AD2", "AD3", "AD4", "AD5", "AD6",
           "AD7", "AD8", "AD9", "NSC1", "NSC2", "NSC3", "NSC4", "DR1", "DR2", "DR3")

colnames(data1) <- colnames(data2) <- colnames(data3) <- colnames(data4) <- c(1:22)

data1 <- as.data.frame(data1)
data2 <- as.data.frame(data2)
data3 <- as.data.frame(data3)
data4 <- as.data.frame(data4)
data <- rbind(data1, data2, data3, data4)


# ------------------------------------------------------------------------------------
```

```
# ---------- 3. Descriptives ----------------------------------------------------------
# -----------------------------------------------------------------------------------


nrow(data1)          #219
nrow(na.omit(data1)) #218
nrow(data2)          #193
nrow(na.omit(data2)) #183
nrow(data3)          #186
nrow(na.omit(data3)) #175
nrow(data4)          #280
nrow(na.omit(data4)) #280

data1miss<-na.omit(data1)
data2miss<-na.omit(data2)
data3miss<-na.omit(data3)
data4miss<-na.omit(data4)

### Means and standard deviations
means1 <- colMeans(data1, na.rm=T)
sds1 <- as.vector(sapply(data1, sd, na.rm=T))

means2 <- colMeans(data2, na.rm=T)
sds2 <- as.vector(sapply(data2, sd, na.rm=T))

means3 <- colMeans(data3, na.rm=T)
sds3 <- as.vector(sapply(data3, sd, na.rm=T))

means4 <- colMeans(data4, na.rm=T)
sds4 <- as.vector(sapply(data4, sd, na.rm=T))

# Correlations
cor(means1,sds1, method="spearman") # .38
cor(means2,sds2, method="spearman") # -.78
cor(means3,sds3, method="spearman") # -.83
cor(means4,sds4, method="spearman") # .08
cor(means1,means2, method="spearman") # .48 -- lowest
cor(means1,means3, method="spearman") # .59
cor(means1,means4, method="spearman") # .72
cor(means2,means3, method="spearman") # .87 -- highest
cor(means2,means4, method="spearman") # .52
cor(means3,means4, method="spearman") # .67

# -- mean correlation is .64


# Total means across samples
data1sum <- data1miss; data1sum$s <- rowMeans(data1miss)           # mean 1.63, sd 0.89
mean(rowMeans(data1miss)); sd(rowMeans(data1miss))
data2sum <- data2miss; data2sum$s <- rowMeans(data2miss)           # mean 2.54, sd 0.77
mean(rowMeans(data2miss)); sd(rowMeans(data2miss))
data3sum <- na.omit(data3); data3sum$s <- rowMeans(na.omit(data3))  # mean 2.29, sd 0.90
mean(rowMeans(data3miss)); sd(rowMeans(data3miss))
data4sum <- data4miss; data4sum$s <- rowMeans(data4miss)           # mean 1.36, sd 0.78
mean(rowMeans(data4miss)); sd(rowMeans(data4miss))

# compare means
```

```
t.test(data1sum$s, data2sum$s) # t = -10.96, df = 398.73, p-value < 2.2e-16
t.test(data1sum$s, data3sum$s) # t = -7.2991, df = 370.97, p-value = 1.777e-12
t.test(data1sum$s, data4sum$s) # t = 3.6032, df = 432.96, p-value = 0.0003509
t.test(data2sum$s, data3sum$s) # t = 2.7472, df = 341.77, p-value = 0.006328
t.test(data2sum$s, data4sum$s) # t = 16.115, df = 393.43, p-value < 2.2e-16
t.test(data3sum$s, data4sum$s) # t = 11.377, df = 329.6, p-value < 2.2e-16


meansSD_table <- round(cbind(means, sds), digits=2)
sink("Table2MeansSD.txt")
meansSD_table
sink()

# Internal consistency
alpha1 <- psych::alpha(cor_auto(data1)); alpha1$total$std.alpha # 0.9358135
alpha2 <- psych::alpha(cor_auto(data2)); alpha2$total$std.alpha # 0.9241336
alpha3 <- psych::alpha(cor_auto(data3)); alpha3$total$std.alpha # 0.9486286
alpha4 <- psych::alpha(cor_auto(data4)); alpha4$total$std.alpha # 0.9396545
alpha_total <- psych::alpha(cor_auto(data)); alpha_total # std.alpha=0.95


# --------------------------------------------------------------------------------------
# ---------- 4. Estimate networks individually -----------------------------------------
# --------------------------------------------------------------------------------------

### Compute correlations
data1cor <- cor_auto(data1)
data2cor <- cor_auto(data2)
data3cor <- cor_auto(data3)
data4cor <- cor_auto(data4)

### Individual network estimation
network1 <- estimateNetwork(data1, default="EBICglasso", labels = names)
network2 <- estimateNetwork(data2, default="EBICglasso", labels = names)
network3 <- estimateNetwork(data3, default="EBICglasso", labels = names)
network4 <- estimateNetwork(data4, default="EBICglasso", labels = names)

L <- averageLayout(network1,network2,network3,network4)

### Predictibility
# Re-estimate individual networks via mgm
type=rep('g', 22)

fit1 <- mgm(na.omit(data1), type=type , level=rep(1,22), k=2)
pred1 <- predict(fit1, na.omit(data1), error.continuous='VarExpl')

fit2 <- mgm(na.omit(data2), type=type, lev=rep(1,22), k=2)
pred2 <- predict(fit2, na.omit(data2), error.continuous='VarExpl')

fit3 <- mgm(na.omit(data3), type=type, lev=rep(1,22), k=2)
pred3 <- predict(fit3, na.omit(data3), error.continuous='VarExpl')

fit4 <- mgm(na.omit(data4), type=type, lev=rep(1,22), k=2)
pred4 <- predict(fit4, na.omit(data4), error.continuous='VarExpl')

# Average node predictability in each dataset
mean(pred1$error$Error.R2) # .47
```

```
mean(pred2$error$Error.R2) # .47
mean(pred3$error$Error.R2) # .60
mean(pred4$error$Error.R2) # .54
#mean(c(0.47, 0.47, 0.60, 0.54)) # -- 0.522758

#max(network1$graph) # .522
#max(network2$graph) # .605
#max(network3$graph) # .628
#max(network4$graph) # .649

### Plot networks
par(mfrow=c(2,2))
network1G <- plot(network1, layout=L, title="Austria", maximum = 0.65, theme="classic",
pie=pred1$error$Error.R2,
        border.width=2, vsize=10, border.color='#555555', label.color="#555555", color="#EEEEEE",
labels=names)
network2G <- plot(network2, layout=L, title="Scotland", maximum = 0.65, theme="classic",
pie=pred2$error$Error.R2,
        border.width=2, vsize=10, border.color='#555555', label.color="#555555", color="#EEEEEE",
labels=names)
network3G <- plot(network3, layout=L, title="Wales", maximum = 0.65, theme="classic",
pie=pred3$error$Error.R2,
        border.width=2, vsize=10, border.color='#555555', label.color="#555555", color="#EEEEEE",
labels=names)
network4G <- plot(network4, layout=L, title="Lithuania", maximum = 0.65, theme="classic",
pie=pred4$error$Error.R2,
        border.width=2, vsize=10, border.color='#555555', label.color="#555555", color="#EEEEEE",
labels=names)


pdf("FigS1.pdf", width=10, height=10)
par(mfrow=c(2,2))
makeBW(network1G,colorlist = "")
makeBW(network2G,colorlist = "")
makeBW(network3G,colorlist = "")
makeBW(network4G,colorlist = "")
dev.off()


### Save output
#save(data1cor,data2cor,data3cor,data4cor, file="data_cormatrices.RData")
#save(network1,network2,network3,network4, network1G, network2G, network3G, network4G,
file="networks_individual.RData")
#save(fit1,pred1,fit2,pred2,fit3,pred3,fit4,pred4, file="mgm.Rdata")

# ---------------------------------------------------------------------------------------
# ---------- 5. Stability and accuracy for individually estimated networks --------------
# ---------------------------------------------------------------------------------------

### Estimate and save stability and accuracy
boot1a <- bootnet(network1, nBoots = 1000)
boot1b <- bootnet(network1, nBoots = 1000, type = "case")

boot2a <- bootnet(network2, nBoots = 1000)
boot2b <- bootnet(network2, nBoots = 1000, type = "case")

boot3a <- bootnet(network3, nBoots = 1000)
```

```
boot3b <- bootnet(network3, nBoots = 1000, type = "case")

boot4a <- bootnet(network4, nBoots = 1000)
boot4b <- bootnet(network4, nBoots = 1000, type = "case")

### Plot edge weight CI
pdf("FigS1.pdf")
par(mfrow=c(2,2))
plot(boot1a, labels = FALSE, order = "sample")
plot(boot2a, labels = FALSE, order = "sample")
plot(boot3a, labels = FALSE, order = "sample")
plot(boot4a, labels = FALSE, order = "sample")
par(mfrow=c(1,1))
dev.off()

### Plot centrality stability
pdf("FigS2.pdf")
par(mfrow=c(2,2))
plot(boot1b)
plot(boot2b)
plot(boot3b)
plot(boot4b)
par(mfrow=c(1,1))
dev.off()

### Centrality stability coefficient
cs1 <- corStability(boot1b)
cs2 <- corStability(boot2b)
cs3 <- corStability(boot3b)
cs4 <- corStability(boot4b)
cs <- matrix(NA,4,3)
cs[1,1:3] <- round(cs1, digits=2)
cs[2,1:3] <- round(cs2, digits=2)
cs[3,1:3] <- round(cs3, digits=2)
cs[4,1:3] <- round(cs4, digits=2)
colnames(cs) <- c("Betweenness", "Closeness", "Strength")
rownames(cs) <- c("Sample1", "Sample2","Sample3", "Sample4")
sink("TableS1.txt")
cs
sink()

### Edge weights diff test
pdf("FigS3.pdf")
par(mfrow=c(2,2))
plot(boot1a, "edge", plot = "difference", onlyNonZero = TRUE, order = "sample")
plot(boot2a, "edge", plot = "difference", onlyNonZero = TRUE, order = "sample")
plot(boot3a, "edge", plot = "difference", onlyNonZero = TRUE, order = "sample")
plot(boot4a, "edge", plot = "difference", onlyNonZero = TRUE, order = "sample")
par(mfrow=c(1,1))
dev.off()

### Centrality diff test
pdf("FigS4.pdf")
par(mfrow=c(2,2))
plot(boot1a, "strength", order="sample", labels=FALSE)
plot(boot2a, "strength", order="sample", labels=FALSE)
plot(boot3a, "strength", order="sample", labels=FALSE)
```

```
plot(boot4a, "strength", order="sample", labels=FALSE)
par(mfrow=c(1,1))
dev.off()

### Save output
# save(boot1a, file = "boot1a.Rdata")
# save(boot1b, file = "boot1b.Rdata")
# save(boot2a, file = "boot2a.Rdata")
# save(boot2b, file = "boot2b.Rdata")
# save(boot3a, file = "boot3a.Rdata")
# save(boot3b, file = "boot3b.Rdata")
# save(boot4a, file = "boot4a.Rdata")
# save(boot4b, file = "boot4b.Rdata")


# ------------------------------------------------------------------------------------
# ---------- 6. Joint network estimation ----------------------------------------------
# ------------------------------------------------------------------------------------

### Joint estimation using crossvalidation; this is the main analysis of the paper
EGN1 <- EstimateGroupNetwork(list(data1, data2, data3, data4), method="crossvalidation",
                             strategy="sequential", simplifyOutput = FALSE, seed=1337, covfun = cor_auto)

#max(EGN1$network[[1]]) # 0.5344648
#max(EGN1$network[[2]]) # 0.5899133
#max(EGN1$network[[3]]) # 0.5944841
#max(EGN1$network[[4]]) # 0.5899133

pdf("Fig1.pdf", width=10, height=10)
par(mfrow=c(2,2))
g1 <- qgraph(EGN1$network[[1]], layout = L, title = "Austria", maximum = 0.60,  theme="classic",
pie=pred1$error$Error.R2,
            border.width=2, vsize=10, border.color='#555555', label.color="#555555", color="#EEEEEE",
labels=names)
g2 <- qgraph(EGN1$network[[2]], layout = L, title = "Scotland",maximum = 0.60,  theme="classic",
pie=pred2$error$Error.R2,
            border.width=2, vsize=10, border.color='#555555', label.color="#555555", color="#EEEEEE",
labels=names)
g3 <- qgraph(EGN1$network[[3]], layout = L, title = "Wales",maximum = 0.60, theme="classic",
pie=pred3$error$Error.R2,
            border.width=2, vsize=10, border.color='#555555', label.color="#555555", color="#EEEEEE",
labels=names)
g4 <- qgraph(EGN1$network[[4]], layout = L, title = "Lithuania",maximum = 0.60, theme="classic",
pie=pred4$error$Error.R2,
            border.width=2, vsize=10, border.color='#555555', label.color="#555555", color="#EEEEEE",
labels=names)
dev.off()

pdf("Fig1_bw.pdf", width=10, height=10)
par(mfrow=c(2,2))
g1 <- qgraph(EGN1$network[[1]], layout = L, title = "Austria", maximum = 0.60,   theme="gray",
pie=pred1$error$Error.R2,
            border.width=2, vsize=10, border.color='#555555', label.color="#555555", color="#EEEEEE",
labels=names)
g2 <- qgraph(EGN1$network[[2]], layout = L, title = "Scotland", maximum = 0.60,   theme="gray",
pie=pred2$error$Error.R2,
            border.width=2, vsize=10, border.color='#555555', label.color="#555555", color="#EEEEEE",
```

```
labels=names)
g3 <- qgraph(EGN1$network[[3]], layout = L, title = "Wales", maximum = 0.60,  theme="gray",
pie=pred3$error$Error.R2,
            border.width=2, vsize=10, border.color='#555555', label.color="#555555", color="#EEEEEE",
labels=names)
g4 <- qgraph(EGN1$network[[4]], layout = L, title = "Lithuania", maximum = 0.60,  theme="gray",
pie=pred4$error$Error.R2,
            border.width=2, vsize=10, border.color='#555555', label.color="#555555", color="#EEEEEE",
labels=names)
dev.off()


g1_edges <- g1$Edgelist; length(g1_edges$weight) # 108 edges are non zero
g2_edges <- g2$Edgelist; length(g2_edges$weight) # 107 edges are non zero
g3_edges <- g3$Edgelist; length(g3_edges$weight) # 117 edges are non zero
g4_edges <- g4$Edgelist; length(g4_edges$weight) # 113 edges are non zero


### Correlations
# Correlations joint networks with individual networks
cor(getWmat(g1)[lower.tri(getWmat(g1))], getWmat(network1)[lower.tri(getWmat(network1))],
method="spearman")   #0.87
cor(getWmat(g2)[lower.tri(getWmat(g2))], getWmat(network2)[lower.tri(getWmat(network2))],
method="spearman")   #0.88
cor(getWmat(g3)[lower.tri(getWmat(g3))], getWmat(network3)[lower.tri(getWmat(network3))],
method="spearman")   #0.87
cor(getWmat(g4)[lower.tri(getWmat(g4))], getWmat(network4)[lower.tri(getWmat(network4))],
method="spearman")   #0.83

# Correlations joint networks with each other
cor(getWmat(g1)[lower.tri(getWmat(g1))], getWmat(g2)[lower.tri(getWmat(g2))],   method="spearman") #0.69
cor(getWmat(g1)[lower.tri(getWmat(g1))], getWmat(g3)[lower.tri(getWmat(g3))], method="spearman") #0.72
cor(getWmat(g1)[lower.tri(getWmat(g1))], getWmat(g4)[lower.tri(getWmat(g4))], method="spearman") #0.75
cor(getWmat(g2)[lower.tri(getWmat(g2))], getWmat(g3)[lower.tri(getWmat(g3))], method="spearman") #0.73
cor(getWmat(g2)[lower.tri(getWmat(g2))], getWmat(g4)[lower.tri(getWmat(g4))], method="spearman") #0.73
cor(getWmat(g3)[lower.tri(getWmat(g3))], getWmat(g4)[lower.tri(getWmat(g4))], method="spearman") #0.72
#mean(.73,.74,.67,.73,.62,.69) # = .73

### Save output
#save(EGN1,L, g1,g2,g3,g4, file="JointEstimation.RData")

# -------------------------------------------------------------------------------------
# ---------- 7. Centrality for 4 jointly estimated networks -----------------------------
# -------------------------------------------------------------------------------------

### Estimate and plot centrality
cents <- as.data.frame(cbind(scale(centrality(g1)$InDegree),
                         scale(centrality(g2)$InDegree),
                         scale(centrality(g3)$InDegree),
                         scale(centrality(g4)$InDegree)))

cents <- mutate(cents, id=1:22)

colnames(cents)<-c("1", "2", "3", "4", "Symptoms")
cents
cents_long <- melt(cents, id="Symptoms")
cents_long$Symptoms <- as.numeric(cents_long$Symptoms)
```

```
names(cents_long)[2] <- "Datasets"

samples <- c(rep("Austria", 22), rep("Scotland", 22), rep("Wales", 22), rep("Lithuania", 22))
cents_long$Datasets <- samples
str(cents_long)

pdf("Fig2.pdf", width=6, height=4, useDingbats=FALSE)
p3 <-   ggplot(data=cents_long, aes(x=Symptoms, y=value, colour=Datasets)) +
  geom_line() +
  geom_point(shape = 21, fill = "white", size = 1, stroke = 1) +
  xlab(" ") + ylab("Centrality") +
  scale_y_continuous(limits = c(-3, 3)) +
  scale_x_continuous(breaks=c(1:22), labels=names) +
  theme_bw(base_size = 11, base_family = "") +
  theme(panel.grid.minor=element_blank(), axis.text.x = element_text(angle = 60, hjust = 1)); p3
# coord_flip()
dev.off()

pdf("Fig2_bw.pdf", width=6, height=4, useDingbats=FALSE)
p3 <-   ggplot(data=cents_long, aes(x=Symptoms, y=value, linetype=Datasets)) +
  geom_line() +
  geom_point(shape = 21, fill = "white", size = 1, stroke = 1) +
  xlab(" ") + ylab("Centrality") +
  scale_y_continuous(limits = c(-3, 3)) +
  scale_x_continuous(breaks=c(1:22), labels=names) +
  theme_bw(base_size = 11, base_family = "") +
  theme(panel.grid.minor=element_blank(), axis.text.x = element_text(angle = 60, hjust = 1)); p3
# coord_flip()
dev.off()

### Correlations

hist(scale(centrality(g1)$InDegree))
hist(scale(centrality(g2)$InDegree))
hist(scale(centrality(g3)$InDegree))
hist(scale(centrality(g4)$InDegree)) # we can use Spearman correlation because after scaling the histograms look not normal

# How closely is centrality order related across networks
cor(scale(centrality(g1)$InDegree), scale(centrality(g2)$InDegree),  method = "spearman") # correlations between 0.59 and 0.82
cor(scale(centrality(g1)$InDegree), scale(centrality(g3)$InDegree),  method = "spearman")
cor(scale(centrality(g1)$InDegree), scale(centrality(g4)$InDegree),  method = "spearman")
cor(scale(centrality(g2)$InDegree), scale(centrality(g3)$InDegree),  method = "spearman")
cor(scale(centrality(g2)$InDegree), scale(centrality(g4)$InDegree),  method = "spearman")
cor(scale(centrality(g3)$InDegree), scale(centrality(g4)$InDegree),  method = "spearman")

# Correlation centrality individual networks & symptom means
cor(scale(centrality(g1)$InDegree), means1, method="spearman") # -0.11
cor(scale(centrality(g2)$InDegree), means2, method="spearman") # 0.29
cor(scale(centrality(g3)$InDegree), means3, method="spearman") # 0.30
cor(scale(centrality(g4)$InDegree), means4, method="spearman") # 0.14

# Correlation centrality individual networks & symptom standard deviations
cor(scale(centrality(g1)$InDegree), sds1, method="spearman") # 0.14
cor(scale(centrality(g2)$InDegree), sds2, method="spearman") # <0.01
cor(scale(centrality(g3)$InDegree), sds3, method="spearman") # -0.12
```

```
cor(scale(centrality(g4)$InDegree), sds4, method="spearman") # 0.50
#mean(.14,.000001,-.12,.50) # = .14

# Correlation centrality & predictability
cor(pred1$error$Error.R2, centrality(g1)$InDegree) # .87
cor(pred2$error$Error.R2, centrality(g2)$InDegree) # .86
cor(pred3$error$Error.R2, centrality(g3)$InDegree) # .80
cor(pred4$error$Error.R2, centrality(g4)$InDegree) # .90

### Save files
# save(p3, cents, file="centrality.RData")


# ------------------------------------------------------------------------------------
# ---------- 8. Network comparison tests for 4 jointly estimated networks ---------------
# ------------------------------------------------------------------------------------


### First, how similar are polychoric and Pearson?
c1 <- cor(data1miss)
c1b <- cor_auto(data1miss)
cor(c1[lower.tri(c1)],     c1b[lower.tri(c1b)],     method="spearman") #0.987
c2 <- cor(data2miss)
c2b <- cor_auto(data2miss)
cor(c2[lower.tri(c2)],     c2b[lower.tri(c2b)],     method="spearman") #0.981
c3 <- cor(data3miss)
c3b <- cor_auto(data3miss)
cor(c3[lower.tri(c3)],     c3b[lower.tri(c3b)],     method="spearman") #0.988
c4 <- cor(data4miss)
c4b <- cor_auto(data4miss)
cor(c4[lower.tri(c4)],     c4b[lower.tri(c4b)],     method="spearman") #0.992

# since they are very similar, we can probably use the NCT() that is only validated for Pearson, not poylchoric
correlations

### NCT topology
names(data1miss) <- names(data2miss) <- names(data3miss) <- names(data4miss) <- c(1:22)

set.seed(1337)
compare_12 <- NCT(data1miss,data2miss, it=5000, binary.data=FALSE, test.edges=TRUE, edges='all',
progressbar=TRUE)
compare_13 <- NCT(data1miss,data3miss, it=5000, binary.data=FALSE, test.edges=TRUE, edges='all',
progressbar=TRUE)
compare_14 <- NCT(data1miss,data4miss, it=5000, binary.data=FALSE, test.edges=TRUE, edges='all',
progressbar=TRUE)
compare_23 <- NCT(data2miss,data3miss, it=5000, binary.data=FALSE, test.edges=TRUE, edges='all',
progressbar=TRUE)
compare_24 <- NCT(data2miss,data4miss, it=5000, binary.data=FALSE, test.edges=TRUE, edges='all',
progressbar=TRUE)
compare_34 <- NCT(data3miss,data4miss, it=5000, binary.data=FALSE, test.edges=TRUE, edges='all',
progressbar=TRUE)

comparison_topology <- matrix(NA,4,4)
comparison_topology[1,2] <- compare_12$nwinv.pval
comparison_topology[1,3] <- compare_13$nwinv.pval
comparison_topology[1,4] <- compare_14$nwinv.pval
comparison_topology[2,3] <- compare_23$nwinv.pval
```

```
comparison_topology[2,4] <- compare_24$nwinv.pval
comparison_topology[3,4] <- compare_34$nwinv.pval; comparison_topology   # structural invariance

#      [,1]   [,2]    [,3]    [,4]
#[1,]   NA  0.0044  0.0414  0.0158   #  significant p-values with alpha=0.05: 1-2, 1-3, 1-4, 3-4
#[2,]   NA     NA   0.1320  0.0600
#[3,]   NA     NA       NA  0.0486
#[4,]   NA     NA       NA      NA

### quantification of differences: count significantly different edges (total number 231)
sum(compare_12$einv.pvals$"p-value" < 0.05) # 1; 0.004%
sum(compare_13$einv.pvals$"p-value" < 0.05) # 0; 0.000%
sum(compare_14$einv.pvals$"p-value" < 0.05) # 0; 0.000%
sum(compare_23$einv.pvals$"p-value" < 0.05) # 0; 0.000%
sum(compare_24$einv.pvals$"p-value" < 0.05) # 0; 0.000%
sum(compare_34$einv.pvals$"p-value" < 0.05) # 0; 0.000%


# sink("TableS2.txt")
# compare_12$einv.pvals
# compare_13$einv.pvals
# compare_14$einv.pvals
# compare_23$einv.pvals
# compare_24$einv.pvals
# compare_34$einv.pvals
# sink()

### NCT global strength
comparison_strength <- matrix(NA,4,4)
comparison_strength[1,2] <- compare_12$glstrinv.pval
comparison_strength[1,3] <- compare_13$glstrinv.pval
comparison_strength[1,4] <- compare_14$glstrinv.pval
comparison_strength[2,3] <- compare_23$glstrinv.pval
comparison_strength[2,4] <- compare_24$glstrinv.pval
comparison_strength[3,4] <- compare_34$glstrinv.pval; comparison_strength

compare_12$glstrinv.sep # global strength network1: 9.656767    network2: 9.413629
compare_34$glstrinv.sep # global strength network3: 10.129017   network4: 9.888129


#      [,1]   [,2]   [,3]   [,4]
#[1,]   NA   0.3576 0.1094 0.4056  # significant: 2-3, 2-4
#[2,]   NA     NA   0.0136 0.0884
#[3,]   NA     NA     NA   0.3102
#[4,]   NA     NA     NA     NA

#save(compare_12, compare_13, compare_14, compare_23, compare_24, compare_34, comparison_strength,
comparison_topology, file="NCT.RData")


### Additional NCT topology test, not reported in manuscript;
### to avoid low power due to different sample sizes, we sample 5 times equal samples and run the NCT 5 times
each for each comparison
#nrow(data1miss) #218
#nrow(data2miss) #183
#nrow(data3miss) #175
#nrow(data4miss) #280
```

```
#set.seed(1); compare_12X1 <- NCT(sample_n(data1miss, 183),data2miss, it=1000, binary.data=FALSE,
test.edges=TRUE, edges='all', progressbar=TRUE)
#set.seed(2); compare_12X2 <- NCT(sample_n(data1miss, 183),data2miss, it=1000, binary.data=FALSE,
test.edges=TRUE, edges='all', progressbar=TRUE)
#set.seed(3); compare_12X3 <- NCT(sample_n(data1miss, 183),data2miss, it=1000, binary.data=FALSE,
test.edges=TRUE, edges='all', progressbar=TRUE)
#set.seed(4); compare_12X4 <- NCT(sample_n(data1miss, 183),data2miss, it=1000, binary.data=FALSE,
test.edges=TRUE, edges='all', progressbar=TRUE)
#set.seed(5); compare_12X5 <- NCT(sample_n(data1miss, 183),data2miss, it=1000, binary.data=FALSE,
test.edges=TRUE, edges='all', progressbar=TRUE)

#set.seed(1); compare_13X1 <- NCT(sample_n(data1miss, 175),data3miss, it=1000, binary.data=FALSE,
test.edges=TRUE, edges='all', progressbar=TRUE)
#set.seed(2); compare_13X2 <- NCT(sample_n(data1miss, 175),data3miss, it=1000, binary.data=FALSE,
test.edges=TRUE, edges='all', progressbar=TRUE)
#set.seed(3); compare_13X3 <- NCT(sample_n(data1miss, 175),data3miss, it=1000, binary.data=FALSE,
test.edges=TRUE, edges='all', progressbar=TRUE)
#set.seed(4); compare_13X4 <- NCT(sample_n(data1miss, 175),data3miss, it=1000, binary.data=FALSE,
test.edges=TRUE, edges='all', progressbar=TRUE)
#set.seed(5); compare_13X5 <- NCT(sample_n(data1miss, 175),data3miss, it=1000, binary.data=FALSE,
test.edges=TRUE, edges='all', progressbar=TRUE)

#set.seed(1); compare_14X1 <- NCT(data1miss,sample_n(data4miss, 218), it=1000, binary.data=FALSE,
test.edges=TRUE, edges='all', progressbar=TRUE)
#set.seed(2); compare_14X2 <- NCT(data1miss,sample_n(data4miss, 218), it=1000, binary.data=FALSE,
test.edges=TRUE, edges='all', progressbar=TRUE)
#set.seed(3); compare_14X3 <- NCT(data1miss,sample_n(data4miss, 218), it=1000, binary.data=FALSE,
test.edges=TRUE, edges='all', progressbar=TRUE)
#set.seed(4); compare_14X4 <- NCT(data1miss,sample_n(data4miss, 218), it=1000, binary.data=FALSE,
test.edges=TRUE, edges='all', progressbar=TRUE)
#set.seed(5); compare_14X5 <- NCT(data1miss,sample_n(data4miss, 218), it=1000, binary.data=FALSE,
test.edges=TRUE, edges='all', progressbar=TRUE)

#set.seed(1); compare_23X1 <- NCT(sample_n(data2miss, 175),data3miss, it=1000, binary.data=FALSE,
test.edges=TRUE, edges='all', progressbar=TRUE)
#set.seed(2); compare_23X2 <- NCT(sample_n(data2miss, 175),data3miss, it=1000, binary.data=FALSE,
test.edges=TRUE, edges='all', progressbar=TRUE)
#set.seed(3); compare_23X3 <- NCT(sample_n(data2miss, 175),data3miss, it=1000, binary.data=FALSE,
test.edges=TRUE, edges='all', progressbar=TRUE)
#set.seed(4); compare_23X4 <- NCT(sample_n(data2miss, 175),data3miss, it=1000, binary.data=FALSE,
test.edges=TRUE, edges='all', progressbar=TRUE)
#set.seed(5); compare_23X5 <- NCT(sample_n(data2miss, 175),data3miss, it=1000, binary.data=FALSE,
test.edges=TRUE, edges='all', progressbar=TRUE)

#set.seed(1); compare_24X1 <- NCT(data2miss,sample_n(data4miss, 183), it=1000, binary.data=FALSE,
test.edges=TRUE, edges='all', progressbar=TRUE)
#set.seed(2); compare_24X2 <- NCT(data2miss,sample_n(data4miss, 183), it=1000, binary.data=FALSE,
test.edges=TRUE, edges='all', progressbar=TRUE)
#set.seed(3); compare_24X3 <- NCT(data2miss,sample_n(data4miss, 183), it=1000, binary.data=FALSE,
test.edges=TRUE, edges='all', progressbar=TRUE)
#set.seed(4); compare_24X4 <- NCT(data2miss,sample_n(data4miss, 183), it=1000, binary.data=FALSE,
test.edges=TRUE, edges='all', progressbar=TRUE)
#set.seed(5); compare_24X5 <- NCT(data2miss,sample_n(data4miss, 183), it=1000, binary.data=FALSE,
test.edges=TRUE, edges='all', progressbar=TRUE)

#set.seed(1); compare_34X1 <- NCT(data3miss,sample_n(data4miss, 175), it=1000, binary.data=FALSE,
```

```
test.edges=TRUE, edges='all', progressbar=TRUE)
#set.seed(2); compare_34X2 <- NCT(data3miss,sample_n(data4miss, 175), it=1000, binary.data=FALSE, test.edges=TRUE, edges='all', progressbar=TRUE)
#set.seed(3); compare_34X3 <- NCT(data3miss,sample_n(data4miss, 175), it=1000, binary.data=FALSE, test.edges=TRUE, edges='all', progressbar=TRUE)
#set.seed(4); compare_34X4 <- NCT(data3miss,sample_n(data4miss, 175), it=1000, binary.data=FALSE, test.edges=TRUE, edges='all', progressbar=TRUE)
#set.seed(5); compare_34X5 <- NCT(data3miss,sample_n(data4miss, 175), it=1000, binary.data=FALSE, test.edges=TRUE, edges='all', progressbar=TRUE)

# structural invariance
#compare_12X1$nwinv.pval # 0.006
#compare_12X2$nwinv.pval # 0.015
#compare_12X3$nwinv.pval # 0.007
#compare_12X4$nwinv.pval # 0.003
#compare_12X5$nwinv.pval # 0.012

#compare_13X1$nwinv.pval # 0.033
#compare_13X2$nwinv.pval # 0.063
#compare_13X3$nwinv.pval # 0.001
#compare_13X4$nwinv.pval # 0.011
#compare_13X5$nwinv.pval # 0.002

#compare_14X1$nwinv.pval # 0.023
#compare_14X2$nwinv.pval # 0.004
#compare_14X3$nwinv.pval # 0.003
#compare_14X4$nwinv.pval # 0.006
#compare_14X5$nwinv.pval # 0.012

#compare_23X1$nwinv.pval # 0.124
#compare_23X2$nwinv.pval # 0.148
#compare_23X3$nwinv.pval # 0.095
#compare_23X4$nwinv.pval # 0.095
#compare_23X5$nwinv.pval # 0.048

#compare_24X1$nwinv.pval # 0.092
#compare_24X2$nwinv.pval # 0.190
#compare_24X3$nwinv.pval # 0.312
#compare_24X4$nwinv.pval # 0.095
#compare_24X5$nwinv.pval # 0.037

#compare_34X1$nwinv.pval # 0.024
#compare_34X2$nwinv.pval # 0.061
#compare_34X3$nwinv.pval # 0.028
#compare_34X4$nwinv.pval # 0.115
#compare_34X5$nwinv.pval # 0.171

# individual edge weights
#sum(compare_12X1$einv.pvals$"p-value" < 0.01) # 1
#sum(compare_12X2$einv.pvals$"p-value" < 0.01) # 1
#sum(compare_12X3$einv.pvals$"p-value" < 0.01) # 1
#sum(compare_12X4$einv.pvals$"p-value" < 0.01) # 1
#sum(compare_12X5$einv.pvals$"p-value" < 0.01) # 2; mean=1.2

#sum(compare_13X1$einv.pvals$"p-value" < 0.01) # 3
#sum(compare_13X2$einv.pvals$"p-value" < 0.01) # 3
#sum(compare_13X3$einv.pvals$"p-value" < 0.01) # 5
```

```
#sum(compare_13X4$einv.pvals$"p-value" < 0.01) # 4
#sum(compare_13X5$einv.pvals$"p-value" < 0.01) # 2; mean=3.4

#sum(compare_14X1$einv.pvals$"p-value" < 0.01) # 2
#sum(compare_14X2$einv.pvals$"p-value" < 0.01) # 2
#sum(compare_14X3$einv.pvals$"p-value" < 0.01) # 1
#sum(compare_14X4$einv.pvals$"p-value" < 0.01) # 1
#sum(compare_14X5$einv.pvals$"p-value" < 0.01) # 0; mean=1.2

#sum(compare_23X1$einv.pvals$"p-value" < 0.01) # 0
#sum(compare_23X2$einv.pvals$"p-value" < 0.01) # 0
#sum(compare_23X3$einv.pvals$"p-value" < 0.01) # 0
#sum(compare_23X4$einv.pvals$"p-value" < 0.01) # 0
#sum(compare_23X5$einv.pvals$"p-value" < 0.01) # 0; mean=0

#sum(compare_24X1$einv.pvals$"p-value" < 0.01) # 0
#sum(compare_24X2$einv.pvals$"p-value" < 0.01) # 2
#sum(compare_24X3$einv.pvals$"p-value" < 0.01) # 0
#sum(compare_24X4$einv.pvals$"p-value" < 0.01) # 0
#sum(compare_24X5$einv.pvals$"p-value" < 0.01) # 2; mean=4/5

#sum(compare_34X1$einv.pvals$"p-value" < 0.01) # 2
#sum(compare_34X2$einv.pvals$"p-value" < 0.01) # 0
#sum(compare_34X3$einv.pvals$"p-value" < 0.01) # 0
#sum(compare_34X4$einv.pvals$"p-value" < 0.01) # 2
#sum(compare_34X5$einv.pvals$"p-value" < 0.01) # 0; mean=4/5



# ---------------------------------------------------------------------------------------
# ---------- 9. Pool data into one full network ------------------------------------------
# ---------------------------------------------------------------------------------------

names(data1) <- names(data2) <- names(data3) <- names(data4) <- names

### Full dataset
data_full <- rbind(data1,data2,data3,data4)
nrow(data_full)  #878

g_full2 <- (getWmat(g1b) + getWmat(g2b) + getWmat(g3b) + getWmat(g4b)) / 4
cent_full2 <- scale(centrality(g_full2)$InDegree)
cent_full2C <- scale(centrality(g_full2)$Closeness)
cent_full2B <- scale(centrality(g_full2)$Betweenness)

pdf("Fig3a.pdf")
graph_full2 <- qgraph(g_full2, layout=L, border.width=2, vsize=8, border.color='#555555', label.color="#555555", 
color="#EEEEEE",
                    legend.cex = 0.6, theme="classic", labels = names)
dev.off()

pdf("Fig3a_bw.pdf")
graph_full2 <- qgraph(g_full2, layout=L, border.width=2, vsize=8, border.color='#555555', label.color="#555555", 
color="#EEEEEE",
                    legend.cex = 0.6, theme="gray", labels = names)
dev.off()

### Similarity
```

```
cor(getWmat(g_full)[lower.tri(getWmat(g_full))],        getWmat(g_full2)[lower.tri(getWmat(g_full2))],
method="spearman") #0.83
cor(cent_full, cent_full2, method="spearman") #0.93

### Plot centrality
cents2 <- as.data.frame(cent_full2)
cents2 <- mutate(cents2, id = 1:22)
colnames(cents2)<-c("value", "Symptoms")
cents2$Symptoms <- as.numeric(cents2$Symptoms)

pdf("Fig3c.pdf", width=6, height=3)
p4 <- ggplot(data=cents2, aes(x=Symptoms, y=value)) +
  geom_line(color="#555555") +
  geom_point(shape = 21, fill = "white", size = 2, stroke = 1, color="#555555") +
  xlab(" ") + ylab("Centrality") +
  scale_y_continuous(limits = c(-3, 3)) +
  scale_x_continuous(breaks=c(1:22), labels=names) +
  theme_bw() +
  theme(panel.grid.minor=element_blank(), axis.text.x = element_text(angle = 60, hjust = 1)); p4
# coord_flip();
dev.off()

# Construct a network where edges are standard deviations across edge weights of networks
edgeMeanJoint <- matrix(0,22,22)
edgeSDJoint <- matrix(0,22,22)
for(i in 1:22){
  for(j in 1:22) {
    vector  <-  c(getWmat(g1)[i,j],getWmat(g2)[i,j],getWmat(g3)[i,j],getWmat(g4)[i,j])
    edgeMeanJoint[i,j] <- mean(vector)
    edgeSDJoint[i,j] <- sd(vector)
  }
}
min(edgeSDJoint) # 0
max(edgeSDJoint) # .13

edgeSDJoint[5,6]   # TH1 and TH2 0.13
edgeSDJoint[7,8]   # AD1 and AD2 0.10
edgeSDJoint[6,9]   # TH2 and AD3 0.09
edgeSDJoint[1,6]   # RE1 and TH2 0.09
edgeSDJoint[2,6]   # RE2 and TH2 0.08
edgeSDJoint[10,11] # AD4 and AD5 0.08

sum(sort(edgeSDJoint[lower.tri(edgeSDJoint)])>0.08) # 6 edges larger than 0.08

pdf("Fig3b.pdf")
var_joint <- qgraph(edgeSDJoint, layout=L, theme="colorblind", border.width=2, vsize=8, labels = names,
                    border.color='#555555', label.color="#555555", color="#ffffff", edge.color="#666666", maximum=.13,
minimum=.03)
dev.off()



### Save objects
#save(data_full_cor, network_full, g_full, cent_full, g_full2, cent_full2, graph_full2, cents2, var_joint,
file="network_pooled.RData")
```
