# Supplementary material for: A Cross‐Cultural Comparison of ICD‐11 Complex Posttraumatic Stress Disorder Symptom Networks in Austria, the United Kingdom, and Lithuania
Source: J Trauma Stress. 2019 Jan 28;33(1):41–51. doi: 10.1002/jts.22361 (PMC7155025; doi:10.1002/jts.22361)
Supplement: Supplementary file 2 — Figure S1. Stability analysis: Accuracy of edge weights. Bootstrapped confidence intervals (CIs) of the edge weights for the four individually estimated networks, derived from non‐parametric bootstrap (nBoot=1000) analyses using R‐package bootnet (Epskamp et al., 2017). Figure S2. Stability Analysis: Centrality bootstrap. Correlation of the original centrality order with the order of centrality in subsets of the data. Figure S3. Edge weights difference test. Black boxes represent significant differences between edge weights. The test does presently not correct for multiple testing. Figure S4. Centrality difference test. Standardized centrality values are shown in the diagonal, black boxes represent significant differences centrality estimates. The test does presently not correct for multiple testing. Figure S5. Centrality for the four independently estimated networks. Note that only strength had acceptable stability and thus the size of the other estimates should not be interpreted. [file JTS-33-41-s002.docx]

**Supplementary Materials**


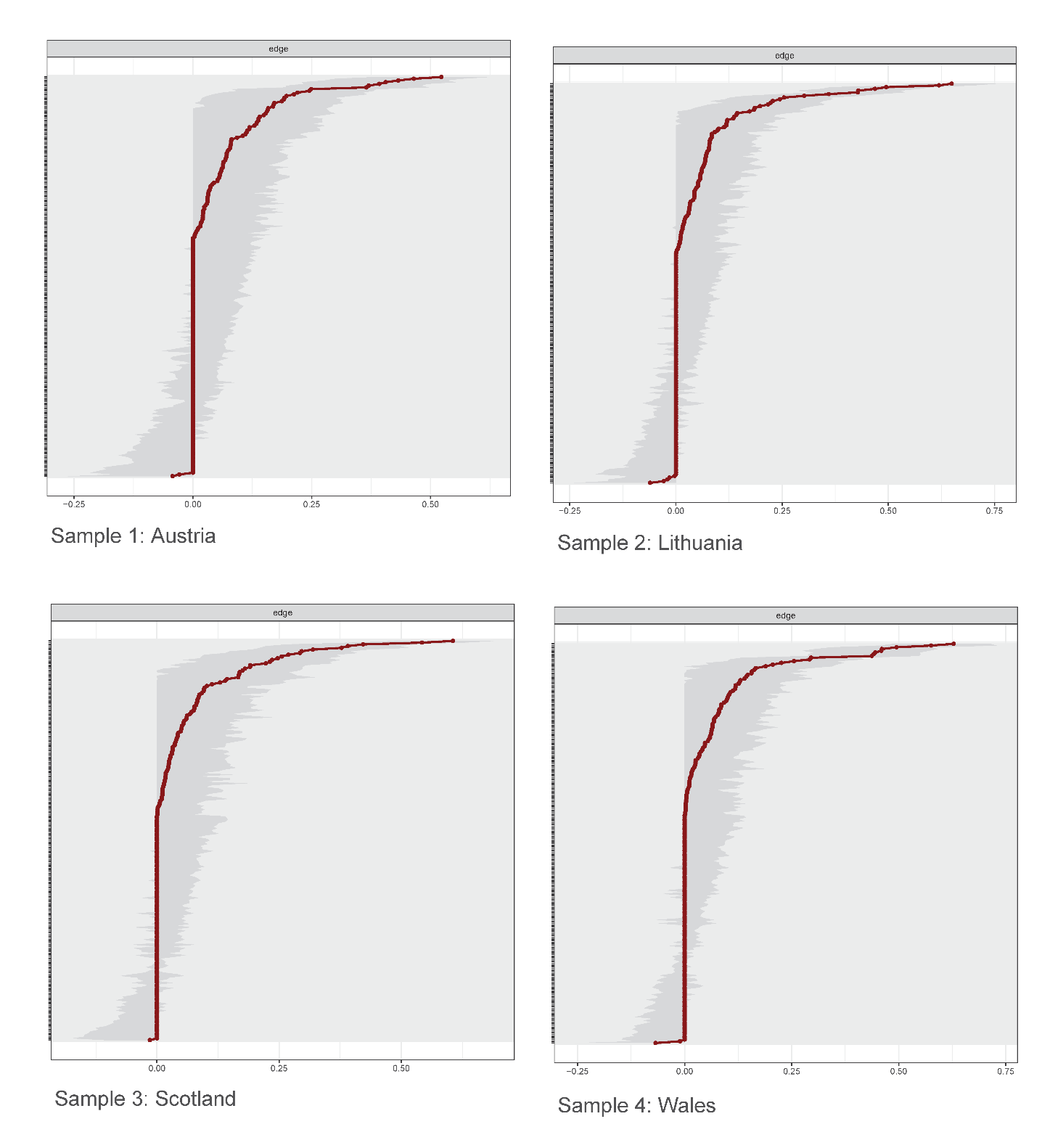
Figure S1. Stability analysis: Accuracy of edge weights. Bootstrapped confidence intervals (CIs) of the edge weights for the four individually estimated networks, derived from non-parametric bootstrap (nBoot=1000) analyses using R-package bootnet (Epskamp et al., 2017). The red line indicates the edge weight values and the grey area the 95% CIs.


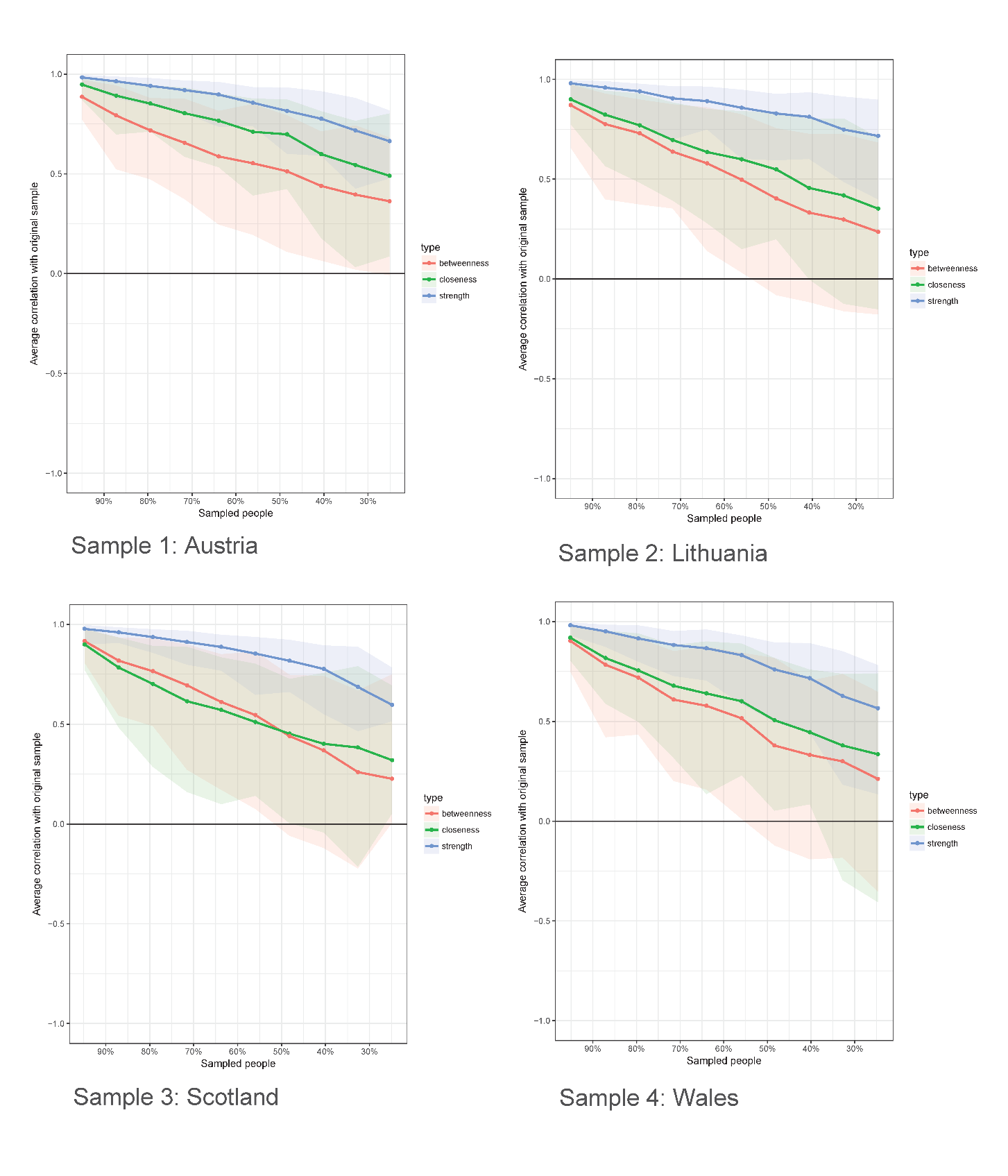
Figure S2. Stability Analysis: Centrality bootstrap. Correlation of the original centrality order with the order of centrality in subsets of the data. The correlation after dropping a substantial number of participants is high for the centrality metric strength, which means that this centrality estimate can be considered stable in all four samples. Only the centrality-stability coefficients for strength was above the suggested threshold of 0.25. The centrality-stability coefficients for the four networks were (betweenness, closeness, strength): Austria (0.05, 0.21, 0.44, resp.), Scotland (0.05, 0.05, 0.52, resp.), Wales (0.05, 0.05, 0.36, resp.), and Lithuania (0.05, 0.05, 0.44, resp.), which implies that only strength should be interpreted.


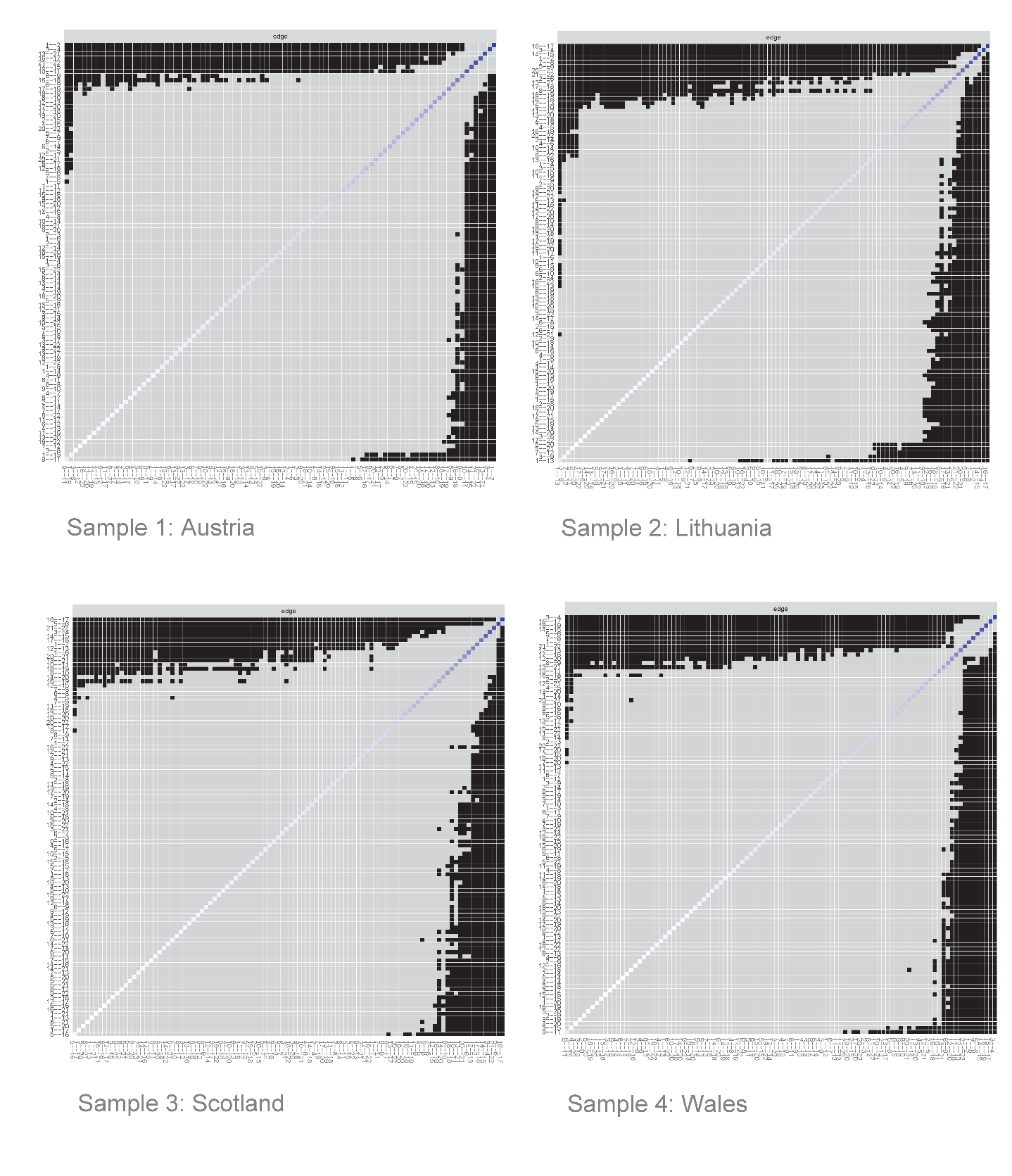
Figure S3. Edge weights difference test. Black boxes represent significant differences between edge weights. The test does presently not correct for multiple testing.


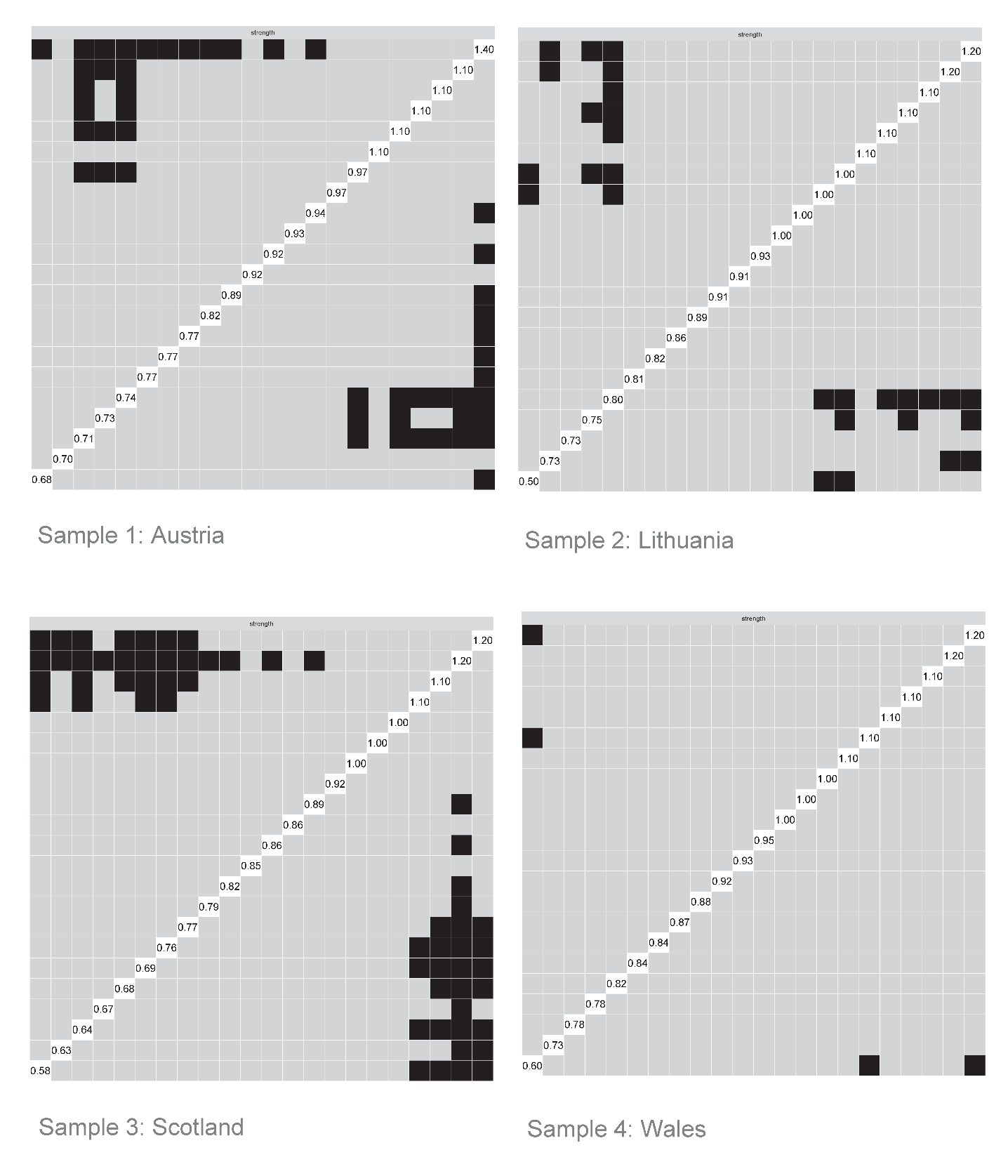
Figure S4. Centrality difference test. Standardized centrality values are shown in the diagonal, black boxes represent significant differences centrality estimates. The test does presently not correct for multiple testing.


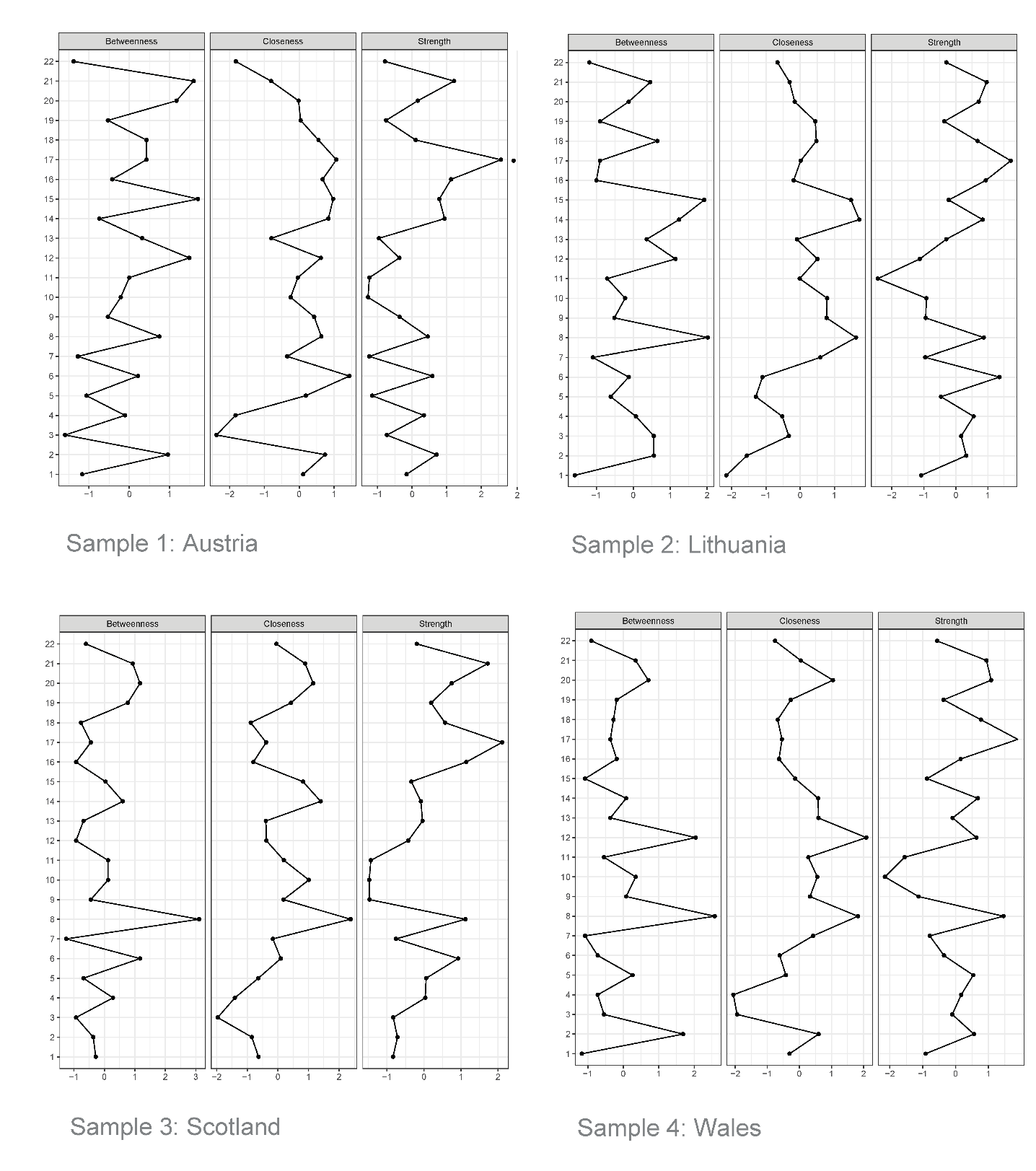
Figure S5. Centrality for the four independently estimated networks. Note that only strength had acceptable stability and thus the size of the other estimates should not be interpreted.
